# *SFRP* Tumour Suppressor Genes Are Potential Plasma-Based Epigenetic Biomarkers for Malignant Pleural Mesothelioma

**DOI:** 10.1155/2017/2536187

**Published:** 2017-12-13

**Authors:** Yuen Yee Cheng, Ellie Mok, Sarah Tan, Catherine Leygo, Chris McLaughlin, A. M. George, Glen Reid

**Affiliations:** ^1^Asbestos Diseases Research Institute (ADRI), University of Sydney, Sydney, NSW, Australia; ^2^School of Life Sciences, University of Technology Sydney, Sydney, NSW, Australia

## Abstract

Malignant pleural mesothelioma (MPM) is associated with asbestos exposure. Asbestos can induce chronic inflammation which in turn can lead to silencing of tumour suppressor genes. Wnt signaling pathway can be affected by chronic inflammation and is aberrantly activated in many cancers including colon and MPM. *SFRP* genes are antagonists of Wnt pathway, and *SFRP*s are potential tumour suppressors in colon, gastric, breast, ovarian, and lung cancers and mesothelioma. This study investigated the expression and DNA methylation of *SFRP* genes in MPM cells lines with and without demethylation treatment. Sixty-six patient FFPE samples were analysed and have showed methylation of *SFRP2* (56%) and *SFRP5* (70%) in MPM. *SFRP2* and *SFRP5* tumour-suppressive activity in eleven MPM lines was confirmed, and long-term asbestos exposure led to reduced expression of the *SFRP1* and *SFRP*2 genes in the mesothelium (MeT-5A) via epigenetic alterations. Finally, DNA methylation of *SFRP*s is detectable in MPM patient plasma samples, with methylated *SFRP2* and *SFRP5* showing a tendency towards greater abundance in patients. These data suggested that *SFRP* genes have tumour-suppresive activity in MPM and that methylated DNA from *SFRP* gene promoters has the potential to serve as a biomarker for MPM patient plasma.

## 1. Introduction

Malignant pleural mesothelioma (MPM) is an aggressive and invariably fatal malignancy associated with asbestos exposure. The exact mechanism by which asbestos exposure leads to MPM carcinogenesis is not yet understood. It is known that asbestos is capable of inducing chronic inflammation and that chronic inflammation is capable of inducing tumour suppressor gene (TSG) silencing which is a driver of cancer. However, a much better understanding of the mechanism by which asbestos exposure leads to MPM is needed, so that the molecular players in the mechanism can be used as new molecular targets for diagnosis and treatment of MPM [1, 2].

It has been proposed that asbestos silicates attract and bind cations and that asbestos needles in the lungs will both retain the ions on the asbestos fibre surface and leach them into the cellular milieu [3], generating reactive oxygen species (ROS) and free radicals that begin the processes of cellular and DNA damage and genotoxicity, driving carcinogenesis [4, 5]. The high iron content of some asbestos fibres, as well as the propensity for asbestos to adsorb iron *in vivo*, has led some authors to suggest that iron-induced Fenton reactions contribute to increased ROS generation, inflammation, and carcinogenesis. It has been shown that asbestos exposure does not directly transform primary human mesothelial cells in tissue culture and instead induces inflammation [6–9]. Chronic inflammation caused by exposure of serosal surfaces to asbestos fibres is likely to represent a central factor in the carcinogenesis of MPM [10, 11].

The mechanism by which asbestos-induced chronic inflammation progresses to MPM carcinogenesis may be through epigenetic changes [10, 11]. A relationship between inflammation and promoter DNA hypermethylation has been documented in many forms of cancer, including MPM [11]. Dysregulation of epigenetic transcriptional control, as well as aberrant promoter DNA methylation and histone modifications in particular, is a fundamental feature of human malignancies [12]. Asbestos exposure may trigger MPM formation via this epigenetic DNA methylation route [10, 13–15], and thus, DNA methylation in MPM is an area of interest for investigation.

Global DNA methylation has been investigated in MPM, and a number of genes were found to be methylated at varying frequencies [10, 16, 17], with the extent of methylation correlating with self-reported asbestos exposure [16] and burden of asbestos fibres in the lung [10]. The level of promoter methylation in MPM cell lines was found to be higher than that found in normal mesothelial cell cultures, and higher methylation status was found in tumours compared with normal mesothelium [10, 16]. The extent of methylation in sarcomatoid MPM was greater for the less differentiated tumours for which prognosis is poorer than that seen in epithelioid MPM tumours, suggesting a link between DNA methylation increase and severity of MPM disease [17]. Methylation-induced silencing of tumour suppressor-like miRs has been observed in MPM, suggesting that aberrant DNA methylation is involved in MPM carcinogenesis [18]. Cell-free methylated promoter DNA from pleural effusion fluid has been used in the diagnosis of malignant pleural effusion for lung cancer [19], and methylated DNA in serum was shown to have prognostic significance in MPM [20]. Therefore, TSG promoter methylation may represent a potential diagnostic and prognostic biomarker for MPM and also a potential therapeutic target. Thus, this study investigated the expression and epigenetic changes to the *SFRP* family of TSGs.

*SFRPs* belong to the family of Wnt pathway antagonists, and the *SFRPs* were found to be silenced in colon, gastric, breast, and lung cancers, with some members silenced in MPM [21–25]. The Wnt signaling pathway is of particular interest because comprehensive studies have shown that this pathway is involved in many cancers. The Wnt pathway is activated in MPM [26], and inhibition leads to apoptosis in gastric cancer cells [21]. Silencing of *SFRP4* and *SRFP5* has been linked to pemetrexed [26] and cisplatin [22] resistance, two drugs used in standard MPM treatment. Epigenetic silencing of the Wnt pathway is well characterized in colon cancer, a cancer known to be related to chronic inflammation. Recently, it was shown that the downregulation of *SFRP* gene family members in gastric and colorectal cancer is mediated by methylation silencing [21, 27]. Thus, downregulation of *SFRP* genes may represent a mechanism of aberrant Wnt signaling activation [21, 27].

In this study, we studied the mRNA expression and methylation of *SFRP*s in a panel of MPM cell lines. We also adopted an *in vitro* asbestos exposure model using the immortalised mesothelial cell line Met-5A and studied the methylation status and mRNA expression of members of the known tumour suppressor *SFRP* gene family. The *SFRP* methylation status in a cohort of 66 MPM patient DNA from FFPE samples was analysed using methylation-specific primers (MSP). Functional significance of *SFRP2* and *SFRP5* was studied in 11 MPM cell lines by cloning *SFRP2* and *SFRP5* into the cells to increase the expression of these genes and measure subsequent retardation in growth and colony formation of MPM cells. Finally, we established a detection method to study DNA methylation of *SFRP* genes in MPM and normal plasma samples with droplet digital PCR (ddPCR).

## 2. Materials and Methods

### 2.1. Cell Lines, Cell Culture, and Treatment

Five MPM cell lines (H2052, H2452, H28, H226, and MSTO) and the immortalised mesothelial cell line MeT-5A were obtained from the American Type Culture Collection (ATCC, Manassas, VA, USA). The primary mesothelioma cell line MM05 [28] was generated at the University of Queensland Thoracic Research Centre (The Prince Charles Hospital, Brisbane). Ren cells [29] were provided by the Laura Moro of the University of Piemonte Orientale A. Avogadro, Novara, Italy. VMC20, VMC23, and VMC40 were generated by The Medical University of Vienna. The primary MPM cell line 1137 was established at ADRI from an MPM patient biopsy. Cells were cultured at 5% CO_2_, 37°C, and 95% humidity in RPMI 1640 (MPM cells) or DMEM (MeT-5A). All media and FBS were from Life Technologies (Carlsbad, CA, USA).

MeT-5A cells were cultured for 3 months in the presence or absence of chrysotile (1 *μ*g/cm^3^). *SFRP1* and *SFRP2* mRNA expression and promoter DNA methylation were analysed by quantitative reverse transcription PCR (RT-qPCR) and quantitative methylation-specific PCR (qMSP) as detailed below. MPM cells were seeded at 5 × 10^5^ cells in a 10 cm^2^ dish and treated 24 h later with 2 *μ*M demethylating agent 5-aza-2′deoxycytidine (decitabine) every 24 h for 5 days. Cells were then harvested for DNA and RNA (detailed below).

### 2.2. Tumour Samples

The tumour samples used in this study are part of a previously reported MPM series to identify biomarkers [30]. All specimens were laser capture microdissected, guided by pathology marked tumour area. Ethics were obtained for this study through the Human Research Ethics Committee at Concord Repatriation General Hospital, Sydney. Written informed consent from all participants was obtained.

### 2.3. Analysis of DNA Methylation

Genomic DNA was extracted from MPM cell lines using the DNA Mini Kit (Qiagen, Valencia, CA, USA) and from MPM FFPE samples using the FFPE DNA Mini Kit (Qiagen) according to the manufacturer's instructions. The methylation status of the *SFRP2* and *SFRP5* promoters in MPM cell lines was determined by MSP or qMSP as described previously [31]. Primers specific for methylated and unmethylated alleles in MSP were as specified previously [21]. CpGenome universal methylated DNA (Millipore, Billerica, MA, USA) was used as a positive control for methylation, and normal buffy coat (BC) from a healthy donor was used as a control for the unmethylated locus in each amplification.

### 2.4. Reverse Transcription and Quantitative Real-Time PCR (RT-qPCR)

Total RNA was extracted from cell lines using Trizol reagent (Life Technologies). Reverse transcription (RT) reactions were performed using 200 ng of total RNA with MMLV first strand cDNA kit (Promega, Madison, WI, USA) following the manufacturer's protocol. The expression of *SFRPs* mRNA was determined by RT-PCR or quantitative real-time PCR using the KAPA SYBR Fast qPCR Master Mix (Sigma) and VII7 QPCR System (Life Technologies). 18S was used as the reference gene. mRNA expression levels of SFRP genes were determined using the 2^−∆∆Cq^ method [32] with normalisation to the 18S gene.

### 2.5. Expression Plasmids and Transfection

The pcDNA3.1(+)*SFRP2* or pcDNA3.1(+)*SFRP5* expression construct was generated by PCR cloning, and the sequence was verified and subcloned into pcDNA3.1-TOPO expression vector (Life Technologies) as previously described [20]. Plasmids were introduced into cells by transfection with X-tremeGENE 9 DNA transfection reagent (Sigma) as per the manufacturer's instructions.

### 2.6. Immunofluorescence of *SFRP2* and *SFRP5* Expression

Cells transfected with an *SFRP2* or *SFRP5* expression construct or empty vector-transfected cells were fixed with 4% paraformaldehyde (Sigma, St. Louis, MO, USA) in PBS for 15 min, washed three times with PBS, and permeabilized with 0.2% Triton X-100 in PBS for 5 min. Fixed cells were blocked with PBS containing 0.1% Triton and 10% fetal bovine serum for 1 h at room temperature. For immunostaining, cells were incubated for 2 h at room temperature with rabbit anti-*SFRP2* or anti-*SFRP5* and mouse anti-*β*-actin antibody at 2.5 *μ*g/mL (Abcam) in blocking solution. After three washes with PBS, cells were incubated for 1 h at room temperature with Alexa Fluor 596-labeled goat anti-rabbit antibody (Life Technologies) and Alexa Fluor 488-labeled goat anti-mouse antibody (Life Technologies). Nuclear counterstaining was performed with 0.5 *μ*g/mL DAPI. Immunostained cells were observed under a fluorescence microscope.

### 2.7. Proliferation Assay

The rate of *in vitro* cell proliferation was assessed by quantifying increases in DNA measured by the SYBR green-based assay. MPM cells were transfected with an *SFRP2* or *SFRP5* expression construct or vector control in 96-well plates, every 24 hrs. One set of plates with medium was removed and the plates were frozen. Relative cell numbers were determined by staining with 200 *μ*L/well of SYBR green I (Life Technologies) as described previously [31]. Proliferation was calculated and presented as a percentage of the intensity of control cells at 120 hrs. Each experiment was performed in triplicate.

### 2.8. Droplet Digital PCR

Primers for the amplification of methylated *SFRP* DNA via MSP were optimized using ddPCR EvaGreen (Bio-Rad) according to the manufacturer's recommendations. DNA isolated from 200 *μ*L plasma samples was bisulfite converted as previously described [21], and 4 *μ*L from a total of 50 *μ*L converted DNA was used as a template for ddPCR. For a 20 *μ*L ddPCR reaction, 2× EvaGreen ddPCR Supermix (Bio-Rad) and primers at a final concentration of 0.2 *μ*M were used. Reactions were dispensed into each well of the droplet generator DG8 cartridge (Bio-Rad). Each oil compartment of the cartridge was filled with 70 *μ*L of droplet generation oil for EvaGreen (Bio-Rad), and approximately 15000 to 20000 droplets were generated in each well with the use of the droplet generator (Bio-Rad QX200). The entire droplet emulsion volume (40 *μ*L) was transferred onto a 96-well PCR plate (Eppendorf). The plate was then heat sealed with a pierceable foil in the PX1 PCR Plate Sealer (Bio-Rad) and placed in the thermocycler (Bio-Rad T1000). The thermal cycling conditions used were 95°C for 5 min; 40 cycles of 95°C for 30 s, 60°C for 30 s, 72°C for 1 min, and a final step at 72°C for 1 min. The reaction mixtures were then held at 4°C until needed. The cycled droplets were read individually with the QX200 droplet-reader (Bio-Rad) and analysed with QuantaSoft droplet reader software, version 1.7 (Bio-Rad). The error reported for a single well was the Poisson 95% confidence interval. The method of no template controls (NTC) was used to monitor contaminations and primer-dimer formation and to determine the cut-off threshold. Normal and plasma sample-positive populations were then used to calculate the positive number detected in the sample, and results were plotted as total copy number detected per sample.

### 2.9. Statistical Analysis

Student's *t*-test was used to compare the difference in proliferation of *SFRP2* or *SFRP5* and control-transfected cells, and *p* < 0.05 was taken as statistically significant for differences between the two groups. Overall survival (OS) was calculated from the time of surgery to the time of death or last follow-up. Methylation status of *SFRP2* and *SFRP5* was used to assess the association of DNA methylation with OS using the Kaplan-Meier method and Cox regression. Statistical calculations were carried out using SPSS version 18.0 for Windows (SPSS Inc., Chicago, IL, USA). A value of *p* < 0.05 was considered statistically significant.

## 3. Results and Discussion

### 3.1. *SFRP* Genes Are Silenced by DNA Hypermethylation in MPM Cell Lines and Tumour Samples

**Figure 1:**
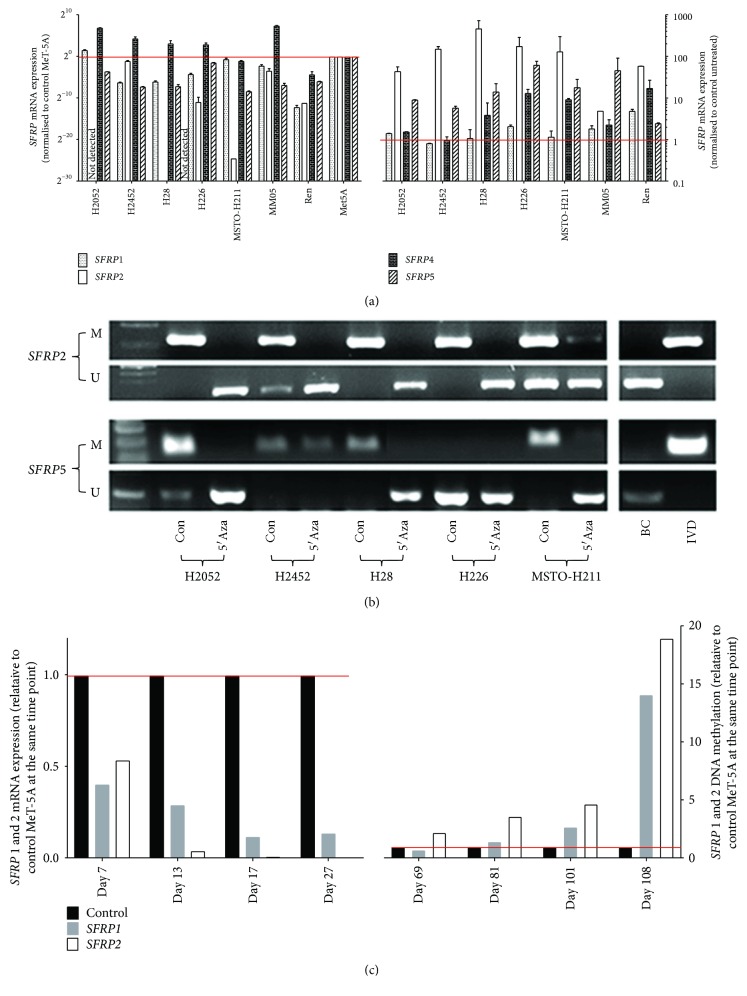
Asbestos-induced downregulation of tumour suppressor gene expression due to DNA methylation. (a) Basal expression and demethylated expression of mRNA of *SFRP* genes were determined by RT-qPCR in 7 MPM cell lines and in MeT-5A. Results were normalized to 18S and are expressed relative to the expression of MeT-5A or control untreated cells. (b) The methylation status of *SFRP2* and *SFRP5* were determined by MSP cell lines. (c) The expression of *SFRP1* was determined by RT-qPCR and DNA methylation by qMSP in MeT-5A cells with or without asbestos exposure. mRNA expression or methylation status was presented as fold change to parental untreated MeT-5A cells. (M = methylated; U = unmethylated, BC = buffy coat isolated from a healthy donor, IVD = universal methylated DNA control).

*SFRP* genes are antagonists of the Wnt-signaling pathway and have been reported to be aberrantly activated in MPM [25, 26, 33]. Some members of the *SFRP* gene family are reported to be silenced via DNA methylation in MPM. Here, we confirm these studies by analyzing *SFRP* mRNA expression levels and DNA methylation status in seven cell lines. We first looked at mRNA expression of *SFRP1*, *SFRP2*, *SFRP4*, and *SFRP5* in MPM cell lines; *SFRP3* was not included as it does not have a distinctive CpG island. We compared the mRNA expression of *SFRP* genes in MPM cell lines to the noncancer MeT-5A and found that for most of our MPM cell lines *SFRP* genes are downregulated (Figure 1(a)). We treated these MPM cell lines with a demethylating agent (decitabine). Results indicated that expression of at least two *SFRP* genes was reactivated in every MPM cell line (Figure 1(a) right), which is a strong indication of DNA hypermethylation. We then analysed DNA methylation status in MPM cells. We narrowed down our focus to *SFRP2* and *SFRP5*, because for most of the MPM cell lines, there was a very low or no baseline detection of *SFRP*2 and *SFRP*5 (Figure 1(a)). Investigating the DNA methylation status of *SFRP2* and *SFRP5* in MPM cells lines revealed that *SFRP*2 and *SFRP*5 were consistently methylated and became unmethylated when treated with decitabine (Figure 1(b)). Our results reconfirm previous publications that *SFRP*s are downregulated through hypermethylation in MPM cells [33, 34].

#### 3.1.1. The Effect of Asbestos Exposure on Tumour Suppressor Genes SFRP1 and SFRP2

We next studied the relationship between chronic asbestos exposure and tumour suppressor gene regulation. We speculated that the mechanism of asbestos-induced carcinogenesis could involve the suppression of tumour suppressor genes by DNA hypermethylation, as it is known that DNA methylation plays a major role in carcinogenesis [21, 31, 35]. The mesothelial cell line MeT-5A was continuously exposed to low-level chrysotile asbestos (1 *μ*g/cm^2^) for 3 months. Low levels of chrysotile were in line with those not inducing apoptosis in previous studies but did induce ROS (data not shown). RNA and DNA were isolated from cells harvested at different time points. We selected *SFRP1* and *SFRP2* for mRNA expression and DNA methylation studies. In the above results, we observed high expression of *SFRP1* in the MeT-5A cell line. We also observed that *SFRP2* was highly upregulated after decitabine treatment. Therefore, we analysed mRNA expression and the DNA methylation status of the *SFRP1* and *SFRP2* genes following chronic asbestos exposure. Prior to asbestos exposure, *SFRP1* and *SFRP2* were highly expressed and unmethylated in MeT-5A. After asbestos exposure, *SFRP1* and *SFRP2* mRNA expression was progressively downregulated from day 7 to day 27 (Figure 1(c)). In these samples, we also observed low levels of DNA methylation (data not shown), and these methylation results were not significantly different between asbestos treatment and controls. We then measured the DNA methylation status of *SFRP1* and *SFRP2* in later samples by qMSP. Our qMSP results for *SFRP1* and *SFRP2* showed increased methylation from day 81 and becoming highly methylated by day 108 (Figure 1(c)).

This is the first time that exposure of MeT-5A nonmalignant cells to asbestos has been shown to cause downregulated mRNA expression and increased DNA methylation of *SFRP1* and *SFRP2*. Although asbestos is a known carcinogen and exposure is highly linked to development of mesothelioma, DNA methylation of *SFRP* genes has not previously been explored. Previous research hypothesised a link between chronic inflammation elicited by asbestos fibres in the mesothelial cavity, damaged scar tissue induced by the chronic inflammation, and inhibition by asbestos-induced ROS of repair of the scar tissue [36, 37]. Our results confirm our hypothesis that asbestos exposure induces an increase in DNA methylation of the promoter of the *SFRP1* and *SFRP2* tumour suppressor genes and downregulation of mRNA of those genes.

### 3.2. *SFRP2* and *SFRP5* Are Hypermethylated in MPM Tumour Samples

**Figure 2:**
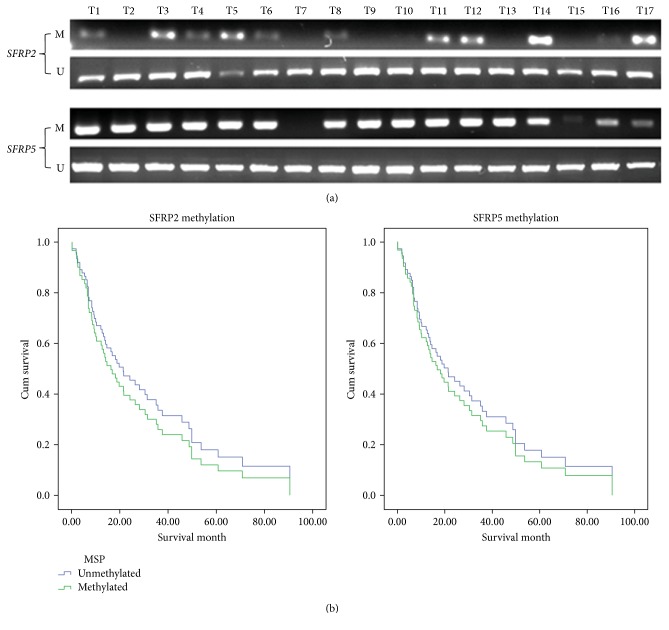
*SFRP2* or *SFRP5* methylation in MPM FFPE samples and overall survival. (a) MSP of *SFRP2* and *SFRP5* using FFPE samples, shown on the gel are representative results. (b) Kaplan-Meier analyses of *SFRP2* (left) and *SFRP5* (right) using methylation results from FFPE samples.

We next analysed the methylation status of *SFRP2* and *SFRP5* in 66 MPM patient samples because these two genes were the most frequently downregulated and methylated in our MPM cell lines. Representative methylation results are shown in (Figure 2(a)). Out of a total of 66 MPM samples, we found that 56% (40 out of 66) and 70% (46 out of 66) have methylation of *SFRP2* and *SFRP5*, respectively, with both *SFRP2* and *SFRP5* methylation detected in 44% (29 out of 66) samples. Methylation of *SFRP2* and *SFRP5* was not detected in samples from healthy normal donors. The effect of *SFRP2* or *SFRP5* DNA methylation on overall survival of MPM patients was assessed using the Kaplan-Meier method (Figure 2(b)). The Cox regression analysis indicated an association between overall survival and *SFRP2* (*p* = 0.06) and *SFRP5* (*p* = 0.56) DNA methylation status in our sample series. The lack of a significant correlation between overall survival of *SFRP2* and *SFRP5* DNA methylation in MPM contrasts with the situation in colorectal cancer, where *SFRP1* and *SFRP2* methylation has potential as an early diagnostic epigenetic biomarker [38].

### 3.3. The Functional Effects of *SFRP2* and *SFRP5* in MPM Cell Invasion and Colony Formation

**Figure 3:**
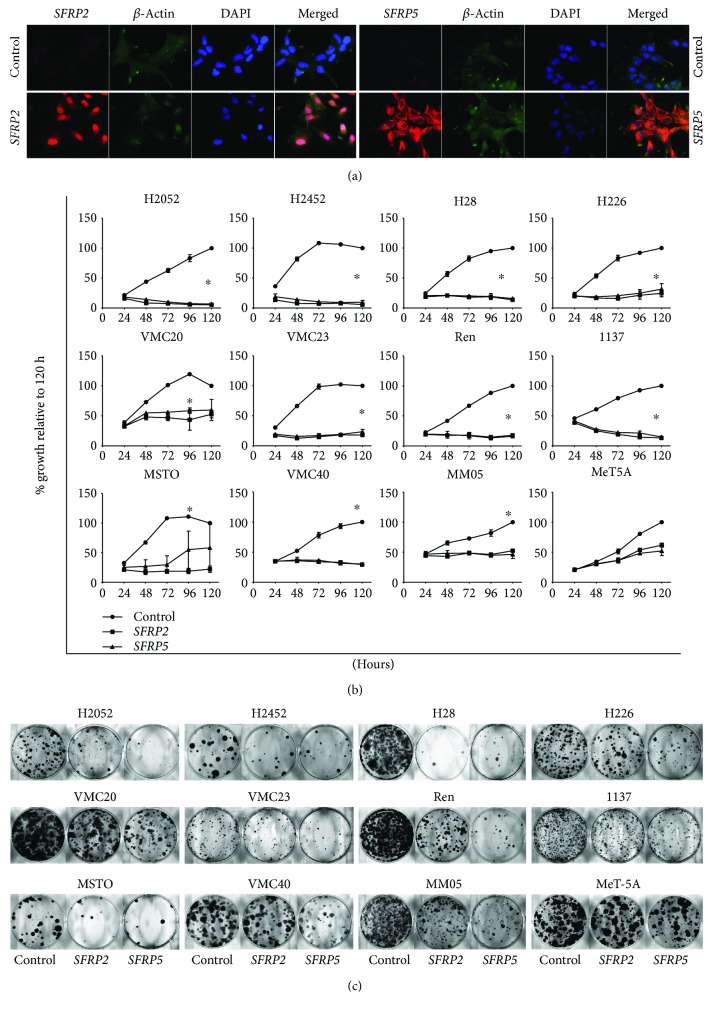
*SFRP2* or *SFRP5* re-expression in MPM cell lines inhibits cell growth and colony formation. (a) Protein re-expression of *SFRP2* and *SFRP5* was confirmed with immunofluorescence *SFRP2* or *SFRP5* red or *β*-actin in green and DAPI nuclear staining in blue. (b) 11 MPM cell lines and MeT-5A were transfected with pcDNA3.1 or pcDNA3.1-*SFRP2* or pcDNA3.1-*SFRP5*, and plates were harvested every 24 hrs for a total of 120 hrs. Cell proliferation was determined by SYBG assay, significant difference between *SFRP2* and *SFRP5* to control as ∗ with *p* < 0.05. (c) The clonogenic potential was assessed by plating 2500 transfected cells per 96 wells and then transferring to a 6-well plate at 24 hrs posttransfection, then incubated for a further 10–14 days. A representative picture from three independent experiments is shown.

Previous research has reported that the *SFRP* antagonists of the Wnt pathway are potential tumor suppressor genes and are frequently silenced and methylated in many cancers [21, 33, 34, 39]. In our study, we show that long-term exposure of asbestos in an *in vitro* model leads to downregulation of *SFRP* tumour suppressor gene expression and this downregulation is via DNA hypermethylation of their promoter regions. Our results for promoter methylation of *SFRP2* and *SFRP5* in MPM suggested that they are downregulated in MPM by hypermethylation and thus may have tumour suppressor potential in MPM. To investigate this, we cloned the ORF of both genes and transfected constructs containing these genes into MPM cells. Confirmation of overexpression of *SFRP2* and *SFRP5* is shown in Figure 3(a). In all 11 MPM cell lines tested, both *SFRP2* and *SFRP5* separately suppressed MPM cell growth compared to control transfections (Figure 3(b), *p* < 0.05). The immortalised MeT-5A cell line showed much less suppression when transfected and this was not significant (Figure 3(c), *p* = 0.07). Ectopic expression of *SFRP2* or *SFRP5* also inhibited the ability of MPM cells to form colonies (Figure 3(c)). Our results confirm that *SFRP2* and *SFRP5* are tumour suppressors of mesothelioma, similar to reports by others [33, 34].

### 3.4. Methylated *SFRP*2 and *SFRP*5 Promoter DNA Is Detectable in MPM Patient Plasma

**Figure 4:**
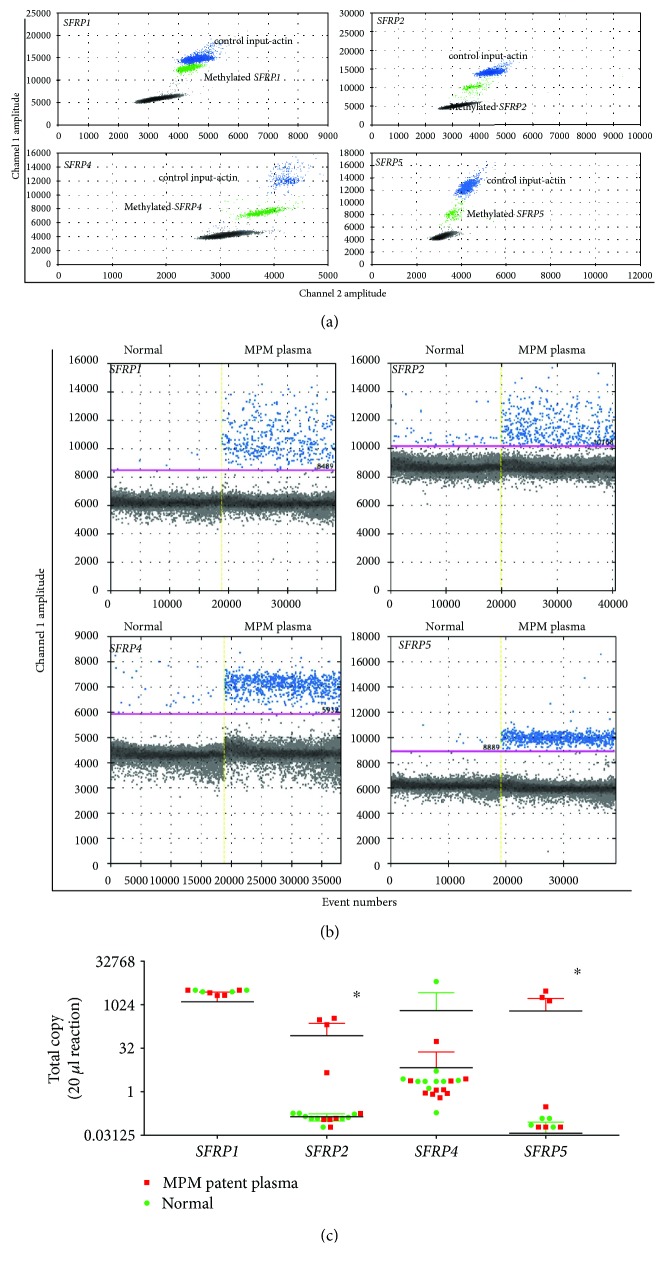
Detection of *SFRPs* in noninvasive plasma samples using droplet digital PCR. (a) Primers for confirmation of methylation of *SFRP* genes using cell lines as control with ddPCR EvaGreen assay. (b) Representative ddPCR results using noninvasive MPM plasma samples methylated or unmethylated fragments were detected using the same primer sets from Figure 4(a). (c) *SFRP* methylation in MPM and normal healthy control plasma was tested using the same ddPCR primer sets and conditions; result output was presented as total copy number detected per 20 *μ*L ddPCR reaction. ddPCR results showed significant (*p* < 0.05^∗^) separation of normal (green) and MPM plasma samples (red) of *SFPR2* and *SFRP5*.

As *SFRP2* and *SFRP5* are frequently methylated in MPM tumour samples, we speculated that the methylated DNA might be released by tumour cells and detectable in plasma. We employed recently available droplet digital PCR technology because it allows quantitative and sensitive detection of nucleic acids. We first optimized ddPCR condition with cell line samples (Figure 4(a)) and then applied this optimized method to the analysis of patient plasma samples (Figure 4(b)). Using the positive population shown in Figure 4(b), we then measured methylation status of *SFRP*s in samples from 10 MPM patients and 10 age-matched controls. We were able to detect methylated DNA from *SFRP2* and *SFRP5* with both showing a distinctive cut-off between patient and healthy normal plasma (Figure 4(c)). We were also able to detect *SFRP1 and SFRP4* methylation in plasma samples; however, there was no distinctive separation of patients and controls. There are many studies reporting detection of biomarkers in noninvasive samples from cancer patients, including sputum (lung cancer) [40], urine (bladder cancer) [41, 42], plasma (breast cancer) [43], and stool (colon cancer) [44, 45]. Although we have demonstrated that methylated *SFRPs* can be detected in plasma of MPM patients, the small sample size means that we are not able to make a definitive conclusion about whether we can use this finding as a diagnostic marker for MPM. These promising early data require validation in a larger series of samples from MPM patients and controls.

## 4. Conclusion

Our gene regulation, DNA methylation, cell growth, and colony formation results indicate that *SFRP2* and *SFRP5* both act as tumour suppressors of MPM and are silenced by DNA hypermethylation. *SFRP1* and *SFRP2* gene expression was downregulated by prolonged asbestos exposure in immortalised noncancer mesothelial cells. We also show that methylation of *SFRP2* (56%) and *SFRP5* (70%) is common in patient samples. The noninvasive detection of *SFRP2* and *SFRP*5 in blood plasma samples demonstrates the potential of using DNA methylation status as a noninvasive epigenetic biomarker for MPM.
